# Cell Communication in a Coculture System Consisting of Outgrowth Endothelial Cells and Primary Osteoblasts

**DOI:** 10.1155/2014/320123

**Published:** 2014-04-22

**Authors:** David Paul Eric Herzog, Eva Dohle, Iris Bischoff, Charles James Kirkpatrick

**Affiliations:** Institute of Pathology, University Medical Center, Johannes Gutenberg University, Langenbeckstraße 1, 55101 Mainz, Germany

## Abstract

Bone tissue is a highly vascularized and dynamic system with a complex construction. In order to develop a construct for implant purposes in bone tissue engineering, a proper understanding of the complex dependencies between different cells and cell types would provide further insight into the highly regulated processes during bone repair, namely, angiogenesis and osteogenesis, and might result in sufficiently equipped constructs to be beneficial to patients and thereby accomplish their task. This study is based on an *in vitro* coculture model consisting of outgrowth endothelial cells and primary osteoblasts and is currently being used in different studies of bone repair processes with special regard to angiogenesis and osteogenesis. Coculture systems of OECs and pOBs positively influence the angiogenic potential of endothelial cells by inducing the formation of angiogenic structures in long-term cultures. Although many studies have focused on cell communication, there are still numerous aspects which remain poorly understood. Therefore, the aim of this study is to investigate certain growth factors and cell communication molecules that are important during bone repair processes. Selected growth factors like VEGF, angiopoietins, BMPs, and IGFs were investigated during angiogenesis and osteogenesis and their expression in the cultures was observed and compared after one and four weeks of cultivation. In addition, to gain a better understanding on the origin of different growth factors, both direct and indirect coculture strategies were employed. Another important focus of this study was to investigate the role of “gap junctions,” small protein pores which connect adjacent cells. With these bridges cells are able to exchange signal molecules, growth factors, and other important mediators. It could be shown that connexins, the gap junction proteins, were located around cell nuclei, where they await their transport to the cell membrane. In addition, areas in which two cells formed gap junctions were found.

## 1. Introduction

Cell communication is conducted in five different ways: endocrine, paracrine, autocrine, electric signalling, and direct cell-cell contacts. A soluble factor or mediator released in the blood stream can reach cells far away from the source, which is called endocrine signalling. When neighbour cells interact, they often release mediators in a paracrine manner to influence the surrounding cells. A number of released mediators can also affect the producing cell itself. This is termed autocrine signalling. Electric signalling occurs in the central nervous system and direct cell-cell contacts are formed by so-called gap junctions, small protein tunnels [1], which consist of different connexin isoforms providing the exchange of ions, small molecules, or potentials [2–4]. Several thousand gap junctions are either located at the same plasma membrane area, called gap junction plaques [5], or are located in the endoplasmic reticulum awaiting their transport to the plasma membrane [6]. In vascular cells, connexins 43, 40, and 37 are the most abundant isoforms [7–10], whereas connexin isoform 43 clearly dominates bone cells [11–15].

The field of tissue engineering aims to provide innovative strategies to compensate for “*the loss or the failure of an organ or tissue, one of the most frequent, devastating, and costly problems in human health care*” [16]. Therefore, bone tissue engineered constructs should afford the key elements of functional and long-lasting bone constructs: mechanical strength, substrates for osteoid formation, sufficient porosity to permit angiogenesis and vasculogenesis, proper vascular network, bone cell migration, controlled degradation to nontoxic products to accommodate the expanding tissue, and controlled inflammation due to high biocompatibility [17–19]. Several studies have shown that the combination of cells of endothelial and osteogenic origin improves both angiogenesis and osteogenesis [20]. It has been controversially discussed which type of endothelial cell should be used for* in vitro* and* in vivo* experiments. Late outgrowth endothelial cells (OECs) can be easily isolated from the peripheral blood mononuclear cell fraction and they possess a high proliferation capacity as well as a strong tendency to form capillary tubes both* in vitro* and* in vivo* [21–24]. It has already been shown that angiogenesis and osteogenesis can be highly improved in a bidirectional way by combining OECs with primary osteoblasts (pOBs) [25, 26], making OECs predestined for bone tissue engineering strategies.

Angiogenesis and osteogenesis are prerequisites for the formation of a functional and perfused tissue engineered construct. Therefore, involved growth factors and mediators during these processes are very important. In our coculture system, essential growth factors were analysed in order to gain better understanding of their contribution to angiogenesis and osteogenesis. Well-known proangiogenic and osteogenic growth factors like* vascular endothelial growth factor A* (VEGF-A), angiopoietins,* platelet-derived growth factor B* (PDGF-B), and* transforming growth factor β* (TGF-*β*) as well as* insulin-like growth factors* (IGFs) and* bone morphogenetic proteins* (BMPs) were examined during the course of cocultivation by comparing two different cultivation time points, 1 and 4 weeks of cocultivation. In addition, by using an indirect coculture system consisting of pOBs and OECs, this study should also evaluate which cell type produces which growth factor. An additional focus of the study was to analyse direct cell-cell interaction via connexins and gap junctions. As well as localizing gap junctions we hoped to establish how connexins behave in the course of coculture growth and development.

## 2. Materials and Methods

### 2.1. Isolation of Outgrowth Endothelial Cells (OEC)

Outgrowth endothelial cells are isolated from the mononuclear cell fraction of peripheral blood buffy coats by ficoll/histopaque separation [26], which is used to separate blood into its components. Therefore, peripheral blood was diluted 1 : 2 in phosphate-buffered saline (PBS) containing 0.5% fetal calf serum (FCS) and 2 mM ethylene diamine tetra-acetic acid (EDTA) to prevent cells from clotting and subsequently centrifuged for 35 min at 400 g (without braking) with histopaque placed at the bottom of a 50 mL tube. After centrifugation 3 different components become visible, from the bottom-up erythrocytes, mononuclear cells, and plasma. The mononuclear cell fraction was separated, washed several times in PBS, and cultured in endothelial cell growth medium-2 (EGM-2) with supplements from the kit, 5% fetal calf serum, and 1% penicillin/streptomycin on collagen-coated plates (35 *μ*g/mL). A total number of 5 ∗ 10^6^ cells per well were seeded on a 24-well plate. After three to four weeks the first OECs with their typical, endothelial cell-like cobblestone morphology and expansion capacity appeared in the culture. OECs were then trypsinized and expanded on fibronectin-coated 24-well plates (10 *μ*g/mL) over several passages at a splitting ratio 1 : 2. Passage numbers of OECs used for this study ranged from passage 8 to 18.

### 2.2. Isolation of Human Primary Osteoblasts

Human primary osteoblasts (pOBs) were isolated from human bone fragments from healthy donors according to an established protocol approved by the responsible Ethical Commission [27]. Collagenase type IV at a concentration of 1 mg/mL was added, several washing steps with PBS followed, and the enzymatically digested bone fragments were subsequently placed on 6-well plates in Dulbecco's modified Eagle medium containing 20% FCS and 1% penicillin/streptomycin (DMEM-Ham F12). During the first two to four weeks cells were fed every day until the plates reached subconfluence. The cells were then transferred to T75 culture flasks and cultured with DMEM-Ham F12 containing only 10% FCS and 1% penicillin/streptomycin. Cells were passaged in a ratio 1 : 2 using accutase. The present study used cells from different donors up to the third passage.

### 2.3. Coculture Consisting of Primary Osteoblasts and Outgrowth Endothelial Cells

Cocultures of OECs and pOBs were seeded on Thermanox coverslips (12 mm in diameter). Primary osteoblasts were seeded first at a density of 300,000 cells/well in a fibronectin-coated 24-well plate followed by seeding of 200,000 OEC/well 24 hours later. Cells were cocultivated in EGM-2 with supplements from the kit, including low VEGF concentrations (2 ng/mL, according to the manufacturer), 5% FCS, and 1% penicillin/streptomycin for different time periods. For each coculture experiment at least three different donors were used. Control pOB- and OEC-monocultures were cultivated in EGM-2 with supplements from the kit, in addition to 5% FCS and 1% penicillin/streptomycin.

### 2.4. Indirect Cocultures Seeded on Transwells

The use of a Transwell filter system allows different cell types in a coculture system to take up and secrete growth factors and signalling molecules on both sides of a transmembrane filter, their basal, and their apical surface. It is thus possible to promote the metabolic activities of these two cell types within a coculture system. 100,000 primary osteoblasts were seeded at the lower surface and 66,000 OECs were seeded at the upper surface of a polycarbonate transmembrane filter in a Transwell filter system in a 24-well plate (pore size 0.4 *μ*m; 0.588 cm^2^/filter) coated with fibronectin (10 *μ*g/mL) to gain insight into the origin of several growth factors produced by the different cell types in the coculture. Cells were fed with EGM-2 with supplements from the kit, 5% FCS, and 1% penicillin/streptomycin and cultured for different time periods. Culture supernatants as well as cell lysates of OEC and pOB were collected separately from the Transwell filter system and then used for further experiments.

### 2.5. Cryostat Sectioning

Cell layers of cocultures consisting of OEC and pOB were snap frozen in liquid nitrogen and sectioned at a thickness of 10 *μ*m using a cryostat (Leica Microsystems, Wetzlar, Germany). Samples were stored at −20°C until use for immunohistochemical analysis. For immunofluorescent staining the sections were first thawed at room temperature before performing the staining procedure as described in the following section.

### 2.6. Paraffin Sectioning

Cocultures of primary osteoblasts and outgrowth endothelial cells were seeded on the upper surface of a fibronectin-coated transmembrane filter in a Transwell filter system. After 14 days of cultivation membranes were fixed with 3.7% PFA for 10 minutes and subsequently washed with PBS. The fixed membranes were cut into appropriate sizes and placed in embedding cassettes. The samples were dehydrated for paraffin embedding in an ascending alcohol series (70%, 80%, 95%, and 100%), each for 1 hour. After an incubation step in xylene for an additional hour, membranes were embedded in paraffin blocks and cut into 4 *μ*m sections and stained for Hematoxylin and eosin** (**HE-stain). For immunofluorescent staining of paraffin sections the sections were rehydrated in a descending ethanol series (100%, 95%, 80%, and 70%) and rinsed in distilled water before staining according to the protocol described below.

### 2.7. 3D Matrigel-Based Angiogenesis Assay

Matrigel Basement Membrane Matrix (Becton Dickinson Labware, Bedford, UK) was thawed on ice and diluted 1 : 2 in cold EBM-2 cell culture medium with supplements from the kit and 5% FCS. 100,000 primary osteoblasts (pOBs) and 66,000 outgrowth endothelial cells (OECs) were resuspended in 50 *μ*L liquid Matrigel/EBM-2 mixture and added to the well of a 96-well plate. The Matrigel Basement Membrane Matrix was allowed to solidify for 30 min at 37°C. Subsequently, 100 *μ*L EBM-2 cell culture medium was added. After 20 h or 40 h of incubation at 37°C in an atmosphere of 5% CO_2_ and 95% air, the cocultures were imaged using a phase-contrast microscope (Biozero, Keyence, Neu-Isenburg, Germany).

### 2.8. Immunofluorescent Staining

Immunofluorescent staining cells were fixed with 3.7% paraformaldehyde (PFA) (Merck, Darmstadt, Germany), washed three times with PBS, and then permeabilized for 5 minutes using 0.1% Triton-X in PBS. Cells were washed again with PBS before being incubated with different primary antibodies diluted in 1% bovine serum albumin (BSA)/PBS for 45 minutes at room temperature: CD31 (dilution 1 : 50; Dako, Hamburg, Germany) and Cx43 (dilution 1 : 250 (mouse)/1 : 400 (rabbit); Millipore, Australia). After washing three times with PBS, cells were incubated with fluorescently labelled secondary antibodies (Alexa; Molecular probes, MoBiTec, Göttingen, Germany), diluted 1 : 1,000 in 1% BSA in PBS, for 45 minutes in darkness at room temperature. Finally, cell nuclei were counterstained with 1 *μ*g/mL Hoechst and cells were mounted with Gelmount (Biomeda, Foster City, CA). The stained samples or frozen sections were examined using a confocal laser scanning microscope (LeicaTCS-NT) (Leica Microsystems, Wetzlar, Germany).

### 2.9. Quantitative Real-Time Polymerase Chain Reaction (Qreal-Time PCR)

RNA isolation was performed using RNeasy Mini Kit according to the manufacturer's protocol (Qiagen, Hilden, Germany). One *μ*g of extracted RNA was transcribed into complementary DNA (cDNA) according to a standard protocol using Omniscript Reverse Transcription Kit (Qiagen, Hilden, Germany). Quantitative real-time PCR, enabling the quantification of relative gene expression, was performed using SYBR green DNA-binding fluorescent dye. 12.5 *μ*L of QuantiTect SYBR Green PCR Master Mix, 2.5 *μ*L of QuantiTect SYBR Green primer assay (VEGF-A, Ang1, Ang2, PDGF-BB, TGF-*β*, IGF-1, IGF-2, ALP, BMP-2, BMP-4, Cx37, Cx40, Cx43; all provided by Qiagen, Hilden, Germany), 6 *μ*L of RNase free water, and 4 *μ*L of cDNA (1 ng/*μ*L) were used for one reaction. Quantitative real-time PCR was performed in triplicate with the following cycler program: 95°C 15 min, denaturation step: 94°C 15 sec, annealing step: 55°C 30 sec, elongation step: 72°C 35 sec, and dissociation: 95°C 15 sec, 60°C 1 min, and 95°C 15 sec; 40 cycles were performed in total. Glycerin-aldehyde-3-phosphate (GAPDH) or ribosomal protein 13A (RPL13A) was taken as endogenous standards and relative gene expression was determined using the ΔΔCt method. Gene expression was compared by setting control cultures to 1 (reference value) as indicated in the relevant figures.

### 2.10. RT^2^ Profiler PCR Array System: Polymerase Chain Reaction Array (PCR Array)

The RT^2^ profiler PCR array system is a pathway-focused gene expression screening method using quantitative real time PCR. RT^2^ profiler PCR arrays contain a panel of 96 primer sets per single 96-well plate for a researched set of 84 relevant pathway-focused genes plus 5 different housekeeping genes. Angiogenesis and osteogenesis RT^2^ profiler PCR array systems were performed with 2 ng cDNA from different experiments according to the manufacturer's protocol. For one reaction 12.5 *μ*L 2x SABiosciences RT^2^ qPCR Master Mix, 1 *μ*L cDNA, and 11.5 *μ*L RNase free water were used. The following cycler program was applied: 95°C 10 min, denaturation step: 95°C 15 sec, annealing step: 60°C 1 min, elongation step: 72°C 35 sec, dissociation: 95°C 15 sec, 60°C 1 min, and 95°C 15 sec; 40 cycles were performed in total. Ribosomal protein 13A (RPL13A) was taken as an endogenous standard and relative gene expression was determined using ΔΔCt method. Gene expression was compared by setting control cultures to 1 (reference value) as indicated in the relevant figures.

### 2.11. Enzyme-Linked Immunosorbent Assay (ELISA)

Culture supernatants from differently treated cells were collected and the concentration of different growth factors was measured using ELISA DuoSets (R&D Systems, Wiesbaden, Germany). ELISA was performed in triplicate according to the manufacturer's protocol. A streptavidin-HRP (horseradish-peroxidase) colorimetric reaction was used to visualize protein concentrations. The optical density of each well was measured using a microplate reader (GENios plus, TECAN, Crailsheim, Germany) at a wavelength of 450 nm. Results are depicted as absolute values as indicated in the relevant figures.

### 2.12. Statistical Analysis

Data are represented as mean values ± standard deviation of the mean. Data distribution was checked with the Shapiro Wilk test. Statistical significance was assessed using the paired Students* t*-test (bilateral *P* value *P < 0.05) and MS-Excel (Microsoft Office; Microsoft, München, Germany) when data could be assessed as normally distributed. Nonnormally distributed data were statistically analysed using the nonparametric Wilcoxon test.

## 3. Results

### 3.1. Cocultures of OECs and pOBs Revealed a Positive Effect on the Cellular Organization of OECs into Angiogenic Structures

**Figure 1:**
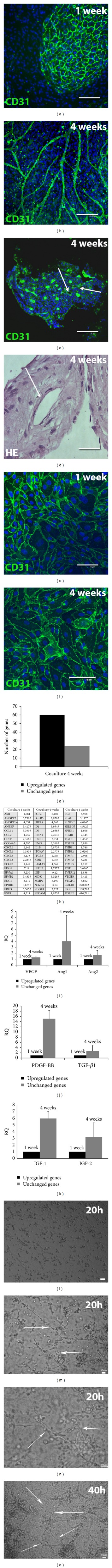
Angiogenesis in cocultures. OEC/pOB cocultures were cultivated for one week (a) and four weeks (b) and for four weeks (c, d)—using cryostat sections—revealing several vessel-like angiogenic structures. Cultures were stained with CD31 to detect endothelium and vessel wall. OEC monocultures were cultivated for one week (e) and four weeks (f) revealing the absence of angiogenic structures. A relevant upregulation of several genes associated with angiogenesis could be detected using a PCR array system (g, h). The relative mRNA expression of the most important growth factors involved in angiogenesis (i–k) was examined and compared after two time points. It was shown that a longer cultivation time led to a better established microvessel system and an upregulation of growth factor mRNA. One week values were set as control (=1.0). (l–o) 3D-Matrigel angiogenesis assay comparing angiogenic behaviour OEC monoculture (L) with cocultures consisting of pOB and OEC (m–o) at different cultivation time points. Scale bars: (a–c) = 150 *μ*m; (d–f) = 75 *μ*m; (l–o) = 50 *μ*m. *n* = 3.

**Figure 2:**
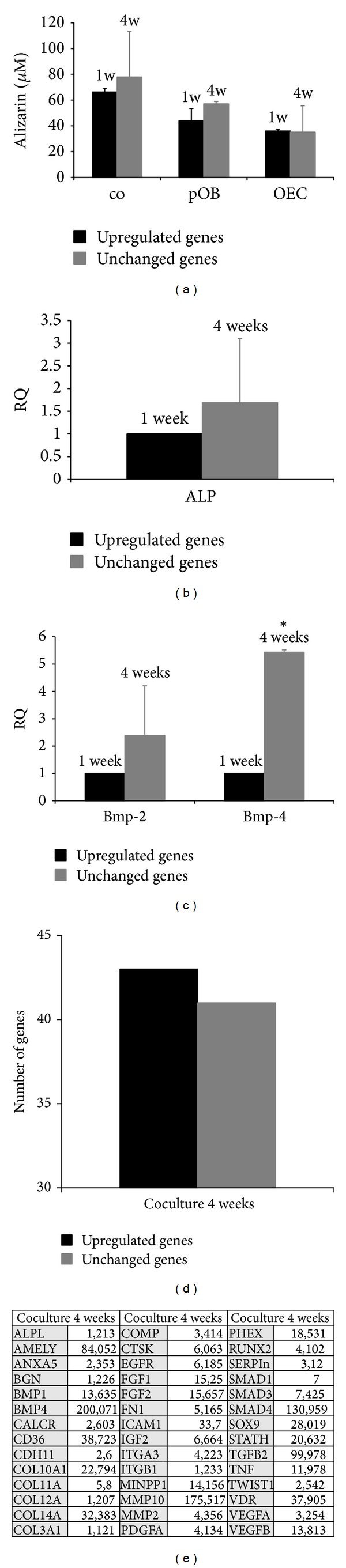
Calcification and osteogenesis. (a) Cocultures (co) and monocultures (pOB and OEC) were compared using alizarin red staining (*μ*M/mg protein) at two different time points (one week versus four weeks) revealing a higher amount in the longer cultivated cocultures and pOBs. ALP (b), BMP-2, and BMP-4 (c) mRNA expression was determined via quantitative real-time RT PCR in cocultures after a cultivation time of one week and four weeks. A relevant upregulation of several genes connected with osteogenesis could be detected using a PCR array system (d, e). It was shown that cocultures after four weeks express a higher amount of ALP, BMP-2, and BMP-4 mRNA than cocultures after one week. One week values were set as control (=1.0) (*P < 0.05), *n* = 3.

To verify the angiogenic potential of the coculture model, outgrowth endothelial cells were cocultured with primary osteoblasts and analysed using immunofluorescent staining for the endothelial marker PECAM (CD31) after 4 weeks of cocultivation and compared to the 1 week coculture (Figure 1(a)). After 4 weeks in coculture, OECs formed numerous microvessel-like structures, reminiscent of a prevascular network, as depicted in Figure 2(b). In the coculture system with pOBs, endothelial cells appeared elongated and formed intercellular contacts and luminar structures, as demonstrated by CD31 immunofluorescent staining of cryostat sections (Figure 1(c)). This formation of a vascular lumen by OECs in the coculture could also be observed in paraffin sections of cocultures seeded on a transmembrane filter in a Transwell filter system (Figure 1(d)). In contrast, OECs in monoculture did not form microvessel-like structures, either after 1 week or after 4 weeks of cultivation (Figures 1(e) and 1(f)). In addition, a 3D angiogenesis assay (Figures 1(m)–1(o)) confirmed this positive angiogenic effect of the coculture system when compared to the endothelial monoculturing alone (Figure 1(l)). Due to the fact that the formation of angiogenic structures formed by OECs clearly increased during the course of cocultivation from 1 week to 4 weeks, a human angiogenesis RT^2^ profiler array (PCR array) was used to compare the expression of angiogenesis-related factors after 1 and 4 weeks of cocultivation (Figures 1(e) and 1(f)). The aim was to gain insight into the molecular mechanisms and possible factors that might be responsible for the proangiogenic effect of the coculture on the OEC. In total, 60 genes involved in the process of angiogenesis were upregulated after 4 weeks of cocultivation compared to 1 week, as depicted in the diagram (Figure 1(g)) and listed in detail in the table in Figure 1(h). In addition, quantification of relative gene expression of well-known angiogenic growth factors, such as VEGF, Ang1, Ang2, IGF-1, IGF-2, TGF-*β*1, and PDGF-BB was determined using quantitative real time PCR (Figures 1(i)–1(k)). The expression level of mRNA after one week was set as control (=1.0). The data clearly show an upregulation of all tested growth factors after 4 weeks of cocultivation compared to 1 week in coculture. In accordance with a higher formation of angiogenic structures after 4 weeks of cocultivation, the expression of proangiogenic factors, for example, VEGF, Ang1, and Ang2 increases after 4 weeks of cocultivation. Additionally, PDGF-BB mRNA, an important pericyte recruiter protein, and TGF-*β*1 mRNA were upregulated. Moreover, IGF mRNAs were also found to be upregulated.

### 3.2. Cocultures of OECs and pOBs Promote Osteogenic Differentiation

Calcification and mineralization level were tested using alizarin red staining and quantitative real-time RT-PCR of osteogenic and bone formation markers, including ALP, BMP-2, and BMP-4 in 4-week cocultures and were compared with the 1-week cultures (Figure 2). The longer cultivation time resulted in higher alizarin red levels in cocultures and pOB monocultures (Figure 2(a)). In general, it is evident that cocultures revealed a higher amount of alizarin red than the monocultures after both one and four weeks of cultivation. Accordingly, relative expression of alkaline phosphatase (ALP), an important enzyme of bone tissue metabolism and marker of osteogenic activity and differentiation, was also higher in the long-term cocultures (Figure 2(b)). BMP-2 and BMP-4 are important during bone formation and bone development. The expression of these molecules was examined on the mRNA level, setting the one week coculture level as control (=1.0). BMP mRNA was affected by a longer cultivation time as well: relative expression of BMP-2 and BMP-4 was in part significantly higher (BMP-4) after four weeks of cocultivation time (Figure 2(c)). Additionally, a PCR array system was used to detect other osteogenesis-related genes (Figure 2(d)). Their relative expression after a cultivation period of four weeks compared to a cultivation period of only one week is depicted in Figure 2(e).

### 3.3. Cell-Cell Communication in Cocultures of pOBs and OECs: Origin of Different Growth Factors

**Figure 3:**
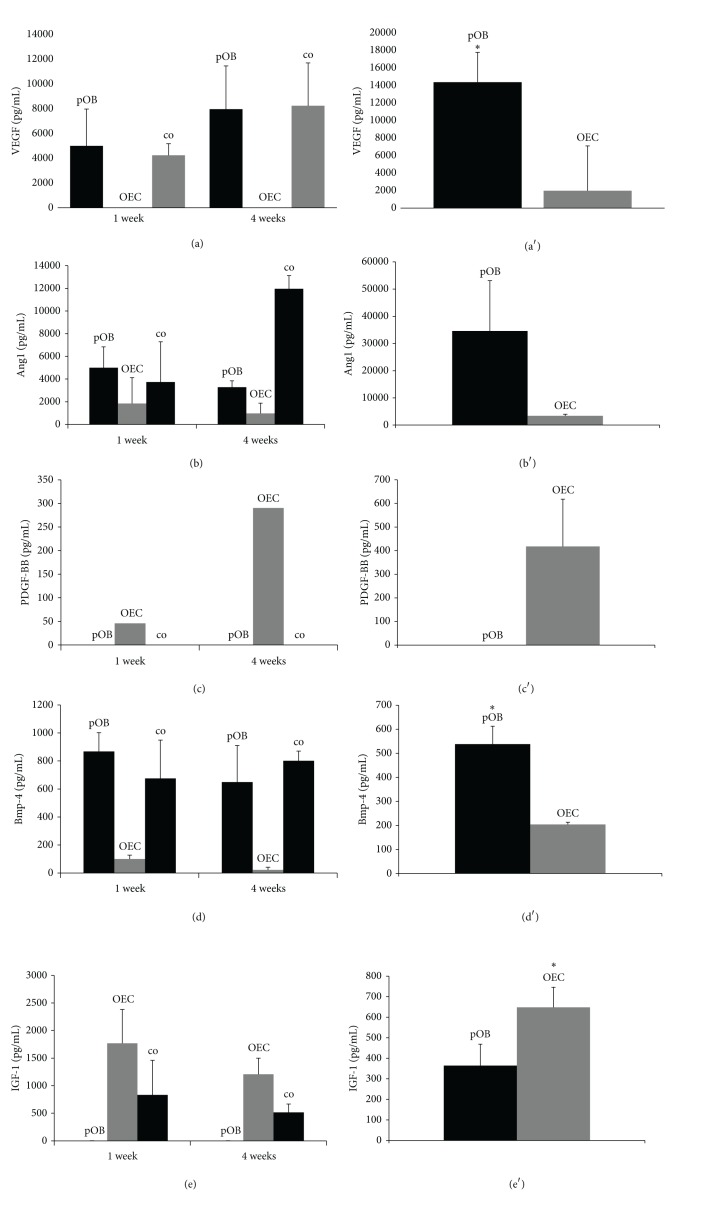
Protein concentration of growth factors contributing to angio- and osteogenesis. Supernatants of direct cocultures, monocultures (left), and four-week cultivated indirect cocultures (right) were collected and VEGF-A (aa′), Ang1 (bb′), PDGF-B (cc′), BMP-4 (dd′), and IGF-1 (ee′) protein concentrations measured to give the distribution throughout the different culture types. The indirect cocultures confirmed the sources of the examined growth factors known from the literature. (*P < 0.05) *n* = 3.

The next object was to determine the source of the involved growth factors: VEGF-A is one of the most important proangiogenic proteins. High VEGF protein levels could be found in direct cocultures as well as in monocultures of primary osteoblasts after both cultivation time points (Figure 3(a)). After four weeks of cultivation, more VEGF-A could be found compared to one week* in vitro*. Practically no VEGF-A could be detected in OEC monocultures. This finding was confirmed by the data of the indirect cocultures, in which the different cell types were separated through a Transwell membrane. Osteoblasts produced significantly (*P < 0.05) more VEGF-A than OECs in indirect cocultures (Figure 3(a′)). Ang1 is an important protein involved in stabilizing and maturing vessels. Ang1 levels correlate with anatomically normal and nonleaky vessel structures. Four-week direct cocultures contained more Ang1 than one-week cocultures and monocultures of pOBs and OECs (Figure 3(b)). Ang1 concentration in both monocultures tended to decrease after four weeks of cultivation compared to one week. Lower levels of Ang1 could be found in OEC monocultures and in OECs of the indirect cultures, whereas pOBs produced high amounts of Ang1 (Figure 3(b′)). PDGF-BB is an important protein during pericyte chemotaxis. It is released by ECs and attracts pericytes and smooth muscle cells to establish a mural layer. The protein was detected in OEC monocultures (Figure 3(c)). PDGF-BB concentration was higher after four weeks of cultivation compared to one week. In addition, indirect cocultures confirmed that PDGF-BB is mainly produced by OECs (Figure 3(c′)). Neither pOB monocultures nor direct cocultures showed any protein concentration. TGF-*β*1 is a very important growth factor involved in different cell activities, including vessel structure maturation and osteogenic cell proliferation. Unfortunately, monocultures and direct cocultures did not produce an appropriate protein concentration, but indirect cocultures showed that pOBs released significantly more TGF-*β*1 than OECs (*P < 0.05) (data not shown). Furthermore, the concentration of BMP-4, an important osteogenic growth factor, was examined in the supernatants of the different cell cultures (Figures 3(d) and 3(d′)). One-week and four-week cocultures and pOB monocultures released high amounts of BMP-4, whereas OECs did not. The indirect cocultures revealed that pOBs clearly produced more BMP-4 than OECs (*P < 0.05) (Figure 3(d′)). In the end, IGF-1 is a protein which has marked effects on nearly all cell types, for example, tissue repair and EC enhancement. The amount of IGF-1 was higher in supernatants of OEC monocultures compared to the cocultures (Figure 3(e)). In addition, IGF-1 concentration decreased in coculture supernatants after four weeks compared to the one-week concentration. Indirect cocultures showed that OECs produced more IGF-1 than pOBs (*P < 0.05) (Figure 3(e′)).

### 3.4. Direct Communication: Connexin Expression in Cocultures of pOBs and OECs

**Figure 4:**
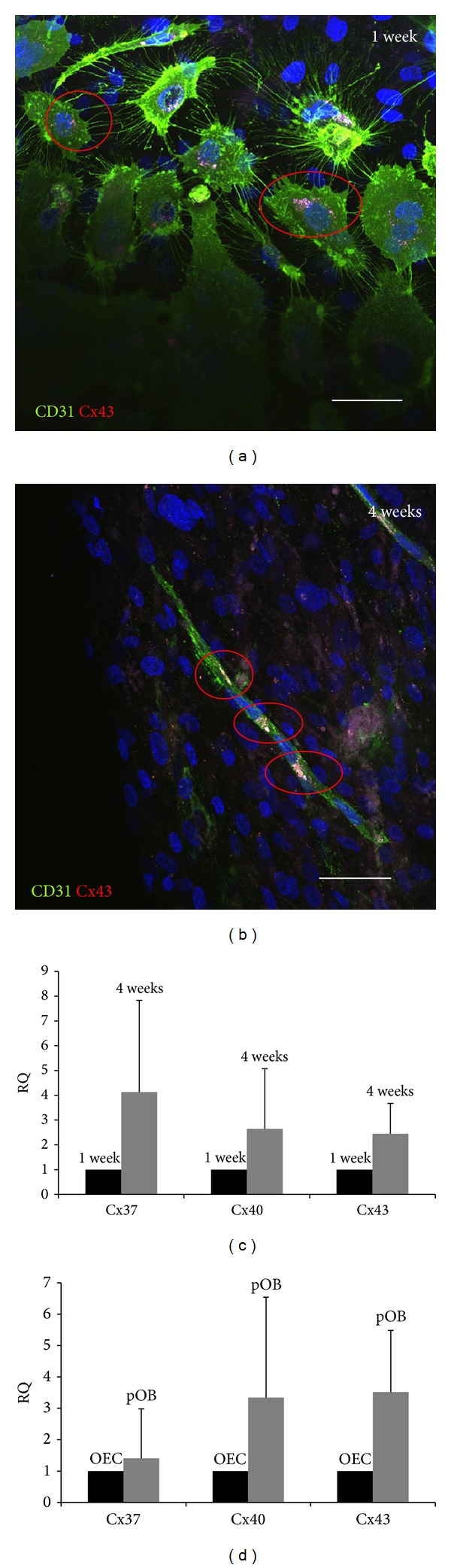
Gap junctional communication in the coculture model system. Several Cx43-positive areas could be detected in our coculture model system after a cultivation time of one week (a) and four weeks (b). Areas such as those shown were scaled up and presumptive gap junctions were found where cells were adjacent. A longer cultivation time led to an upregulation of relative connexin mRNA expression (c) and it seemed that pOBs expressed more connexin mRNA than did OECs (d). One week values (c) were set as control (=1.0) for cocultures and OEC values (d) were set as control (=1.0) to compare pOB and OEC monocultures. Scale bars: 75 *μ*m. *n* = 3.

Connexins are either stored in vesicles around the cell nucleus near to the ER or in the plasma membrane of the cell, contributing to gap junctional intercellular communication. Cx43 expression was analysed in cocultures forming angiogenic structures and stained to give red fluorescence (Figures 4(a)–4(d)). CD-31 was used as endothelial marker and Hoechst dye for visualization of nuclei. A monoclonal Cx43 antibody was selected to reveal connexin localization. In cocultures consisting of pOBs and OECs, few perinuclear Cx43 spots were detectable after one week of coculturing (Figure 4(a)). After four weeks in coculture, when angiogenic structures have been established, many OECs showed typical perinuclear Cx43 conglomerates (Figure 4(b)). In addition, OECs showed Cx43-positive spots at locations where two cells are adjacent, seen between the nuclei of two cells. More mature and expanded microvessels appeared to contain even more gap junctional communication-positive areas in the plasma membrane of the cells. Large connexin conglomerates were located around several nuclei, especially in locations where OECs were merging to some kind of network. Interestingly, there were also a number of nonendothelial cells which contained Cx43-positive areas. The relative gene expression of the three important connexin isoforms during angiogenesis and osteogenesis, namely, Cx37, Cx40, and Cx43, was also assessed in this study. After 4 weeks of cocultivation, an upregulation of all three tested connexin isoforms could be detected. Cx37 expression rose to a 4-fold level and Cx40 and Cx43 to a 2.5-fold level (Figure 4(c)). Although statistical significance was not achieved, a clear trend could be detected. Indirect cocultures might provide the right platform to determine the origin of the connexin isoforms and to obtain some indication of the source of transcription. In general, it was established that connexin mRNA expression is higher in pOBs than in OECs (Figure 4(d)). This difference is relevant in all three connexin isoforms tested: Cx40 expression is 3-fold and Cx43 expression 3.5-fold higher in pOBs compared to OECs.

## 4. Discussion

Cocultures of outgrowth endothelial cells and primary osteoblasts benefit immensely from each other regarding angiogenesis and osteogenesis. After four weeks of coculturing, extensive microvessel networks could be detected compared to a cultivation period of only one week. The promoting effect of osteoblasts on the cellular organization of endothelial cells into tube-like structures in the coculture system is in accordance with reports in the literature [28–31]. Although the detailed mechanisms of the “cellular crosstalk” that controls the angiogenic activation of endothelial cells induced by the coculture are still under investigation, it seems that the coculture with primary osteoblasts provides on the one hand proangiogenic matrix components and on the other hand angiogenic growth factors to promote angiogenic activation of OECs [20, 32]. To analyse the molecular basis of this angiogenic activation of OECs in the coculture system, a human angiogenesis RT² profiler array (PCR array) screening the expression of more than 80 genes involved in the process of angiogenesis was performed after one and four weeks of cocultivation. In total, 60 angiogenesis-related genes were upregulated after four weeks of cocultivation, which was in accordance with the increase in the formation of angiogenic structures in a time-dependent manner, as also reported by Fuchs et al. [32]. The upregulated genes in long term cocultures of OECs and pOBs included growth factors, their receptors, chemokines, cytokines, matrix molecules, and adhesion molecules, for example, vascular endothelial growth factor beta. This could also be confirmed using quantitative real time PCR.

The proangiogenic growth factor, vascular endothelial growth factor A (VEGF-A), for instance, was clearly upregulated after four weeks of cocultivation compared to control cocultures (one week). VEGF-A, known to be the most important mediator of angiogenic activation, has been proposed to be essential for the chemotaxis and differentiation of endothelial progenitor cells, including angioblasts, as well as for endothelial cell proliferation, vascularization via integration of activated endothelial cells into vessel-like structures, and also for remodelling of vascular structures [33–36]. It is mainly produced and secreted by nonendothelial cells, such as pOBs [25, 37]. The indirect cocultures confirmed the osteoblastic origin of VEGF-A in the coculture model system used in this study, whereas OEC monocultures did not show any VEGF-A secretion. This is consistent with previous studies and reports from the literature [37–39]. The four-week protein concentration was even higher, so that expanding pOB monocultures are accompanied by increased concentrations of VEGF-A. Another upregulated proangiogenic molecule after four weeks of cocultivation is angiopoietin 1 (Ang1). Ang1 is expressed and released by perivascular cells in the direction of the endothelium [40]. Its corresponding receptor Tie2 is mainly located on endothelial cells, such as OECs. Ang1 supports vessel formation and vessel wall stability and induces maturation of the growing microvessels [41–43]. In accordance with the literature, in the present study the Ang1 concentration in OEC monocultures was very low, whereas pOB monocultures showed a very high Ang1 protein concentration. The indirect cocultures underline these results and show that considerably more Ang1 is produced in pOBs. Ang1 mRNA was also upregulated after four weeks of cultivation compared to one week, indicating an increasing level of microvessel stabilization and maturation. Platelet-derived growth factor beta was also clearly upregulated in long-term cocultures. The PDGF-B/PDGFR*β* system provides maturation and stabilization of sprouting microvessels. PDGF-B is secreted by endothelial cells, such as OECs, and recruits perivascular cells, which express the PDGFR*β*, in order to stimulate maturation of the vessel wall [44–47]. Examining PDGF-B protein concentration in our model system, the indirect cocultures revealed that PDGF-B production could only be detected in OECs. OEC monocultures secreted only a relatively low PDGF-B amount, whereas PDGF-B concentration increased after four weeks of cocultivation, indicating that culture growth and development seem to correlate with PDGF-B release. Interestingly, protein concentration cannot be measured in cocultures. One explanation may be that cocultures need a large amount of PDGF-B in order to stabilize newly formed microvessels, so that all of the synthesized protein is used and bound to receptors immediately. Another explanation might be that cocultures inhibit the production of PDGF-B in OECs. Recent studies have already shown the importance of PDGF-B throughout bone tissue engineering. Thus, Hollinger et al. have analysed the effect of PDGF-B and *β*-tricalcium phosphate in bone fractures and found a significant contribution to bone repair. Apart from that, recombinant PDGF has already been approved for the repair of periodontal defects [48]. IGF-1 and IGF-2 are two additional growth factors which are involved in angiogenesis [49] and tissue repair [50]. The connection to vessel formation becomes even more understandable when considering the synergistic collaboration of IGF-1 and VEGF [51] in tumour neoangiogenesis. The proteins are reported to be secreted by both osteoblasts and ECs [52]. Indirect cocultures confirmed this observation. Thus, the pOB compartment contained an IGF-1 concentration of ~340 pg/mL and the OEC compartment ~650 pg/mL (*P* < 0.05). OECs seem to have a higher production rate of the protein, which is also supported by monoculture ELISAs. IGF-1 concentration was clearly higher in OEC monocultures after one week of cultivation.

Besides the effect of the coculture system on the angiogenic activation of OEC, the osteogenic potential of the coculture system was also of interest in the scope of this study. In 2009, Fuchs et al. analysed the dynamic processes involved in the differentiation and functionality of both cell types in the coculture system as a function of cultivation time [32]. Due to the fact that different cell types have different demands in terms of culture conditions they tested the influence of the cell culture medium on the osteogenic differentiation of pOB and found an improved calcification of the coculture in endothelial cell growth medium (EGM-2) compared to cultivation in the osteogenic medium, DMEM F-12. In addition, they described a time-dependent upregulation of the osteogenic factors, osteocalcin and osteopontin, in cocultures of pOBs and OECs seeded on silk fibroin, thus indicating an on-going osteoblastic differentiation in the coculture system [53–55]. To screen for effects of the coculture system of pOBs and OECs on osteoblastic differentiation during the course of cocultivation, a human osteogenesis RT² PCR array detecting the expression of 84 genes involved in or related to the process of osteogenic differentiation helped to profile the coculture system in terms of osteogenesis. More than 40 osteogenesis-related genes were found to be upregulated in long term cocultures at four weeks. BMPs are members of the TGF-*β* superfamily and contribute to osteogenic development in cells and tissues* in vivo* and* in vitro*. BMP-2 is involved in endothelial cell development and modulation of several important processes leading to well established vessel networks [56]. BMP-4 induces EC proliferation and migration as well as expression and protein synthesis of VEGFR2 and Tie2 [57], leading to well perfused bone tissue. It is reported that BMP-4 is produced by cells of the osteoblastic lineage and, in fact, indirect cocultures confirmed the osteoblastic origin of this growth factor with statistical significance (*P* < 0.05). This result was accompanied by very low BMP-4 concentrations in OEC monocultures both after one and four weeks of cultivation. In contrast, pOB monocultures showed high concentrations of BMP-4 in the supernatants after both cultivation time points. As expected, high concentration levels of the growth factor could also be detected in direct pOB/OEC cocultures. In addition, ALP expression was slightly higher after a cultivation time of four weeks, although this did not reach statistical significance. This result reveals the osteoplasticity and osteogenic activity of the coculture model system used. ALP is a well-known osteogenic marker protein, because it is essential for the deposition of hydroxyapatite in the bone matrix. High enzyme levels correlate with appropriate osteogenic activity [58–61]. Similar results have been found by Guillotin et al. in 2004, combining* human umbilical vein endothelial cells* (HUVECs) with human osteoprogenitor cells. These authors demonstrated that ALP was upregulated significantly in contrast to monocultures [62]. The calcification level, measured by the alizarin red staining protocol, was also affected positively by the coculture model system; the alizarin red concentration increased and was higher in the cocultures compared to the pOB monocultures at both cultivation time points.

Gap junctional communication is an essential part of cell communication in tissues and cell cultures. It is conducted by connexins, small plasma membrane proteins, which form protein tunnels, so-called gap junctions connecting two adjacent cells. Using this bridge between cells, several signal proteins, metabolites, or other molecules can be exchanged [1, 3, 63]. Each tissue contains its own combination of connexin isoforms. In the vascular system, which consists of ECs and perivascular cells, the most abundant connexin isoform is Cx43, followed by Cx40 and Cx37. Cx43/Cx40, Cx40/Cx40, and Cx43/Cx43 gap junctions contribute to gap junctional communication in the vasculature. Interestingly, Cx43 seems to be expressed mainly in perivascular cells, whereas Cx40 appears to be endothelium-specific. In addition, Cx37 is an important connexin isoform appearing in both cell types of the vascular system, but a participation of Cx37 in gap junctional communication has not been reported [7, 9, 10]. In bone tissue the most abundant connexin isoform is Cx43 [11], which could be confirmed by relative quantification PCR, testing the expression of Cx37, Cx40, and Cx43 in the present coculture model system. After four weeks of cocultivation a relevant upregulation of connexin expression occurred, compared to one-week cultivation time. In this respect, Guillotin et al. showed in 2004 that the combination of HUVECs and human osteoprogenitor cells resulted in a significant upregulation of Cx43 [62]. Gap junctions and connexins are essential for functional bone tissue. Miron et al. have shown that Cx43 overexpression leads to mineralization and osteoblastic differentiation enhancement, whereas Cx43 disruption results in declining levels of ALP expression and insufficient mineralization [64]. Soland and Lampe reported that large connexin conglomerates can be found in the perinuclear region where the endoplasmic reticulum lies [6], whereas established gap junctions are located in so-called gap junctions plaques [5], in bright and punctuate regions, in locations where cells are close together [65]. Selecting Cx43, the typical perinuclear spots could be detected in the present coculture system, thus correlating with the above-mentioned ER-based synthesis of the connexins. In addition, cocultures revealed several Cx43-positive areas in which cells were adjacent. This indicates that Cx43 plays a pivotal role in gap junctional intercellular communication between OECs and pOBs. Longer cultivation time increases this direct cell-cell communication.

In conclusion, this study reveals three key points: (1) coculturing is a superior strategy in terms of vessel formation and bone repair. Both angiogenesis and osteogenesis are improved in the coculture system. Secondly, (2) angiogenesis and osteogenesis appear to be associated with rising levels of growth factors and proteins of different origin. Finally, (3) the study reveals that gap junctional intercellular communication, conducted by connexins, plays a pivotal role in the coculture system. Communication, regulation of cellular processes, and culture development are impossible without connexins. They are important and obligatory mediators between cells throughout angiogenesis and osteogenesis. In general, this study has shown that the orchestra of growth factors and intercellular communication is essential for successful cell culture development.

## Appendix

*RT* ^*2*^ *Profiler TM PCR Array: Human Angiogenesis*

Akt1: V-akt murine thymoma viral oncogene homolog 1
ANGPT2: Angiopoietin 2
ANGPTL4: Angiopoietin-like 4
ANPEP: Alanyl aminopeptidase
CCL11: Chemokine ligand 11
CCL2: Chemokine ligand 2
CDH5: Cadherin5, VE-cadherin
COL4A3: Collagen type 4
COL18: Collagen 18A
CXCL1: Chemokine ligand 1
CXCL3: Chemokine ligand 3
CXCL5: Chemokine ligand 5
CXCL6: Chemokine ligand 6
ECGF1: Endothelial cell growth factor 1 (platelet-derived)
EDG1: Endothelial differentiation, sphingolipid G-protein-coupled receptor 1
EFNA1: Ephrin A1
EFNB2: Ephrin B2
ENG1: Endoglin
EPHB4: Ephrin receptor B4
EREG: Epiregulin
FGF1: Fibroblast growth factor 1
FGF2: Fibroblast growth factor 2
FGFR3: Fibroblast growth factor receptor 3
FIGF: C-fos induced growth factor (vascular endothelial growth factor D)
HIF1A: Hypoxia inducible factor 1, alpha subunit
ID1: Inhibitor of DNA binding 1
ID3: Inhibitor of DNA binding 2
IFNA1: Interferon alpha 1
IFNB1: Interferon beta 1
IFNG: Interferon gamma
IL1b: Interleukin 1 beta
ITGAV: Integrin alpha V
ITGB3: Integrin beta 3
KDR: Kinase insert domain receptor
LAMA5: Laminin alpha 5
LECT1: Leukocyte cell derived chemotaxin 1
LEP: Leptin
MDK: Midkine
MMP2: Matrix metallopeptidase 2
Notch4: Notch homologe 4
PDGFA: Platelet derived growth factor A
PECAM: Platelet endothelial cell adhesion molecule
PGF: Placental growth factor
PLAU: Plasminogen activator
STAB1: Stabilin 1
TGFB1: Transforming growth factor beta 1
TGFB2: Transforming growth factor beta 2
TGFBR: Transforming growth factor beta receptor
THBS1: Thrombospondin 1
THBS2: Thrombospondin 2
TIMP1: TIMP metallopeptidase inhibitor 1
TIMP2: TIMP metallopeptidase inhibitor 2
TIMP3: TIMP metallopeptidase inhibitor 3
TNF: Tumor necrosis factor
TNFA12: Tumor necrosis factor alpha induced protein 2
VEGFA: Vascular endothelial growth factor.

*RT* ^*2*^ *Profiler TM PCR Array: Human Osteogenesis*

ALPL: Alkaline phosphatase
AMELY: Amelogenin
ANXA5: Annexin A5
BGN: Biglycan
BMP4: Bone morphogenetic protein 4
CALCR: Calcitonin receptor
CD36: Thrombospondin receptor
CDH11: Cadherin 11
COL10A: Collagen 10A
COL11A: Collagen 11A
COL12A: Collagen 12A
COL14A: Collagen 14A
COL3A1: Collagen 3A
COMP: Cartilage oligomeric matrix protein
CTSK: Cathepsin K
EGFR: Epidermal growth factor receptor
FGF1: Fibroblast growth factor 1
FGF2: Fibroblast growth factor 2
FN1: Fibronectin 1
ICAM1: Intercellular adhesion molecule 1
IGF2: Insulin like growth factor 2
ITGA3: Integrin alpha 3
ITGB1: Integrin beta 1
MINPP: Multiple inositol polyphosphate histidine phosphatase 1
MMP2: Matrix metallopeptidase 2
PDGFA: Platelet derived growth factor A
PHEX: Phosphate regulating endopeptidase homolog
RUNX: Runt related transcription factor 2
SERPI: Serpin peptidase inhibitor
SMAD1: Smad family member 1
SMAD2: Smad family member 2
SOX9: SRY sex determining region
STATH: Statherin
TGFB2: Transforming growth factor beta 2
TNF: Tumor necrosis factor
TWIST: Twist homolog 1
VDR: Vitamin D receptor
VEGFA: Vascular endothelial growth factor A
VEGFB: Vascular endothelial growth factor B.

## Abbreviations

ALK: Activin receptor-like kinase
ALP: Alkaline phosphatase
Ang: Angiopoietin
ATP: Adenosine triphosphate
bFGF: Basic fibroblast growth factor
BMP: Bone morphogenetic protein
BSA: Bovine serum albumin
cDNA: Complementary deoxyribonucleic acid
CD: Cluster of differentiation
CL: Cytoplasmic loop domain
CT: Carboxyl-terminal domain
Cx43: Connexin isoform 43
DMEM: Dulbecco's modified eagle medium
EC: Endothelial cell
EDTA: Ethylene diamine tetra-acetic acid
e.g.: Exempli gratia
EGM-2: Endothelial cell growth medium 2
ELISA: Enzyme-linked immunosorbent assay
eNOS: Endothelial nitric oxide synthase
EPC: Endothelial progenitor cell
ER: Endoplasmic reticulum
FCS: Fetal calf serum
GJIC: Gap junctional intercellular communication
HIF: Hypoxia-inducible factor
HUVEC: Human umbilical vein endothelial cell
IGF: Insulin-like growth factor
IL: Interleukin
IP3: Inositol trisphosphate
MSC: Mesenchymal stem cell
mRNA: Messenger ribonucleic acid
mTOR: Mammalian target of rapamycin
OEC: Outgrowth endothelial cell
PBS: Phosphate buffered saline
PCR: Polymerase chain reaction.
